# Evaluation of Periodontal Risk Factors with Quantitative Light-Induced Fluorescence Based Fluorescent Plaque Index, in Comparison to Radiographic and Oral Health Habit Scoring: A Retrospective Case Study

**DOI:** 10.3390/s21175774

**Published:** 2021-08-27

**Authors:** Song Hee Oh, Jin-Young Choi, Sae Rom Lee, Seong-Hun Kim

**Affiliations:** 1 Department of Oral and Maxillofacial Radiology, Graduate School of Dentistry, Kyung Hee University, Seoul 02447, Korea; ohbbang50@gmail.com (S.H.O.); saerom0928@gmail.com (S.R.L.); 2 Department of Orthodontics, Graduate School of Dentistry, Kyung Hee University, Seoul 02447, Korea; joyful.ortho@gmail.com

**Keywords:** dental plaque, diagnostic X-ray, panoramic radiograph, periodontitis, quantitative light induced fluorescence

## Abstract

The aim of this study was to evaluate periodontal risk factors with oral health habits and fluorescent plaque index (FPI) using quantitative light-induced fluorescence (QLF) images, and to evaluate their effect on the degree of radiographic bone loss (RBL). Selected were 276 patients over 19 years of age to complete the questionnaire for oral health habit and take QLF images, periapical and panoramic radiographs. Oral health habit score, age, and sex showed a statistically significant correlation with FPI. FPI showed a lower value as the oral health habit score increased and the age decreased. Moreover, females showed lower FPI values than did males. RBL showed a statistically significant positive correlation with age but did not show any correlation with oral health habit scores and sex. There was no correlation between FPI and RBL. The results of this study suggest that the clinical use of QLF allows plaque detection by non-invasive procedures and can aid in a more objective estimation for oral hygiene status.

## 1. Introduction

**Figure 1:**
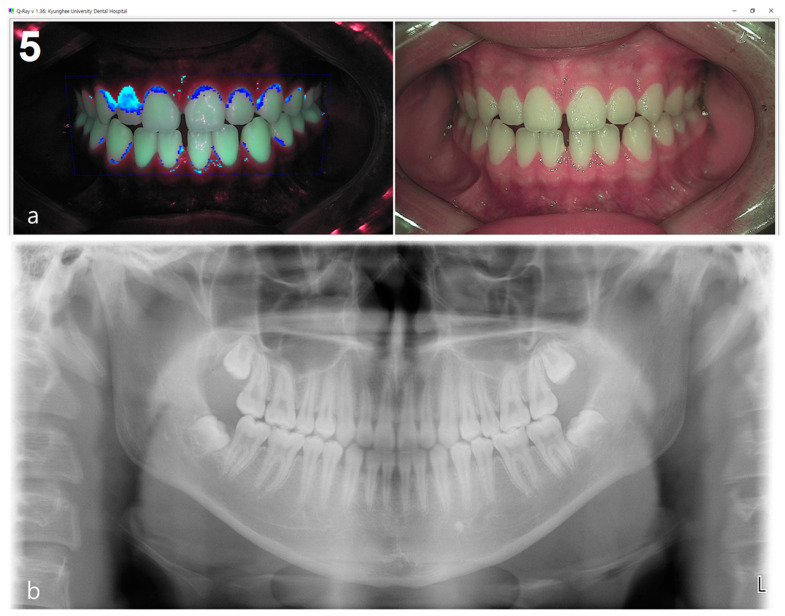
(**a**) Fluorescent plaque index (FPI) score using propriety software of the quantitative light-induced fluorescence (QLF) system was 5 which shows a serious degree of dental plaque pathogenicity, (**b**) while the score of radiographic bone loss (RBL) shown in the panoramic image was 1.1 which was close to normal.

Periodontal diseases, including gingivitis and periodontitis, are some of the most common diseases in humans. Periodontitis is the sixth most prevalent disease worldwide [1]. It is a chronic inflammatory disease that can lead to alveolar bone loss and tooth loss. Alveolar bone loss and tooth loss result from periodontal tissue destruction due to the host’s immune response to the dental biofilm formed on the tooth surfaces. Tooth loss may lead to edentulous and masticatory dysfunction that reduces the oral health-related quality of life in daily life [2]. Periodontitis is a representative oral disease that is related to systemic diseases, such as cardiovascular disease and diabetes [3,4]. Periodontitis affects the individual’s quality of life and self-esteem and imposes huge healthcare costs and socioeconomic impact. In periodontal diseases, the first disease driver is the accumulation of dental plaque (also known as the biofilm) as a result of poor oral hygiene [5]. Dental biofilm is a complex oral microbial ecosystem, where pathogenic bacteria exhibiting periodonto-pathic properties are increased depending on the predominant species present, the metabolites, and host responses [6,7]. Therefore, to prevent and manage periodontal disease, it is important to detect and predict the pathogenic levels of dental plaque. Dental plaque is difficult to observe with the naked eye. Traditionally, to aid with visual examination, a disclosing solution to dye the tooth was used. Currently, this method is not used worldwide because of several disadvantages. First, a disclosing solution stains both the acquired pellicle (which contains few bacteria) and the dental plaque (which is rich in bacteria) non-discriminatively [8,9]. Second, disclosing the plaque visualizes the area covered by dental plaque, but not the thickness or maturity of the plaque. Recently, a new optical method that can replace disclosing agents has been introduced to evaluate dental plaque. Quantitative light-induced fluorescence (QLF) is an optical technique that uses blue visible light with a peak wavelength of 405 nm in combination with a long-pass filter. Using QLF and based on the red fluorescence emission of porphyrins and bacterial metabolites inside the biofilm, the presence and properties of the dental plaque can be determined [10,11,12]. In this way, clinicians can evaluate plaque without using a disclosing solution, and objectively evaluate the dental plaque by quantifying the red fluorescence coverage [13,14]. The intensity of the red fluorescence of QLF is indicative of the dental plaque’s thickness, maturity, and pathogenicity. The classification of periodontitis has been repeatedly revised over the past 30 years based on emerging scientific evidence [15]. In 2017, the American Academy of Periodontology and the European Federation of Periodontology provided a new definition and classification framework for periodontitis, based on a multidimensional staging and grading system [16]. The stage is related to the severity and extent of the current periodontitis, and the grade takes into account the rate at which the disease is progressing [16]. Clinically, periodontal health is indicated by clinical attachment loss, which is assessed by measuring the probing pocket depths and gingival recession. Probing depth measurements are influenced by the probing force, placement, angulation, and tip diameter. These parameters affect the reliability of probing depths [17,18,19]. If clinical attachment loss is not available, radiographic bone loss (RBL) should be used. Radiological imaging is one of the best methods for diagnosing periodontitis [16]. However, detection of initial RBL through X-ray images is limited, and even if detected, it tends to be underestimated [20]. Additionally, it is a factor to consider that X-ray only provides information on bone structure and bone loss, but does not provide information on the current clinical condition (Figure 1) [21,22]. Therefore, in the case of initial periodontal disease, there is a limit in accurately diagnosing with only radiologic images. Periodontal disease tends to be influenced by oral health habits and can be detected early. Disease progression can be prevented through prevention and early treatment. To lower the prevalence of oral diseases such as periodontal disease, prevention is more effective than treatment. Oral care by correcting habits is also necessary. Therefore, we intend to present a more objective clinical guideline for periodontal disease diagnosis and prevention by conducting a comparative analysis of oral health habits using a questionnaire, quantitative fluorescent imaging, and radiographic images among patients who visited the dental health care center for an oral examination.

## 2. Materials and Methods

### 2.1. Study Participants

This study was approved by the Institutional Review Board of Kyung Hee University (IRB No. KH-DT21014), following tenets of the Declaration of Helsinki. Clinical data were collected between July 2020 and June 2021 at the Kyung Hee University Dental Hospital, South Korea. All participants who visited the Kyung Hee Dental Healthcare Center were provided with explanations regarding the objectives and procedures of this study. Patients over 19 years of age and in good health, who provided written agreement to participate were included in the study (N = 276). Forty-four participants were excluded because they presented with orthodontic appliances, esthetic restorations, dental fluorosis, dental caries, stained anterior teeth, or multiple tooth loss due to severe periodontitis. Among the 232 participants (37.0 ± 13.7 years old), 93 were men (33.7 ± 12.3 years old) and 139 were women (39.2 ± 14.0 years old). Participants were not allowed to eat or drink anything, except water, for four hours before the evaluation.

### 2.2. Questionnaire

The questionnaire for oral health management status was modified and supplemented by referring to Yoon et al.’s research tool [23]. The questionnaire included five items, including tooth brushing behavior and the use of oral hygiene products, to investigate oral health habits (Table 1). The oral health habit score was defined as the sum of the scores for each questionnaire item.

### 2.3. QLF Analysis

#### 2.3.1. Acquisition of White-Light and Fluorescent Images

**Figure 2:**
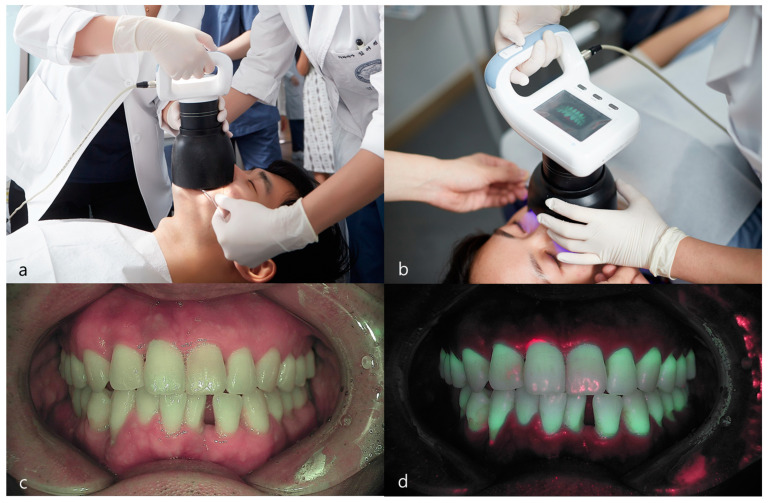
White-light and fluorescent images of the anterior teeth were captured using a Qraycam Pro^®^ (AIOBIO, Seoul, Korea). (**a**,**b**) QLF system was equipped with a metal tube that blocked external light to prevent contamination of the fluorescent image. The participants maintained an edge-to-edge occlusion while capturing the images. (**c**) White-light image; (**d**) fluorescent image.

White-light and fluorescent images of the anterior teeth were captured using a Qraycam Pro^®^ (AIOBIO, Seoul, Korea). The QLF images were captured using ‘anterial’ imaging mode. According to the manufacturer’s instructions, the distance between the specimens and the Qraycam Pro^®^ device was 8 cm. The QLF system was equipped with a metal tube that blocked external light to prevent contamination of the fluorescent image. The participants maintained an edge-to-edge occlusion while capturing the images (Figure 2).

#### 2.3.2. Fluorescent Plaque Index (FPI) Scoring

**Figure 3:**
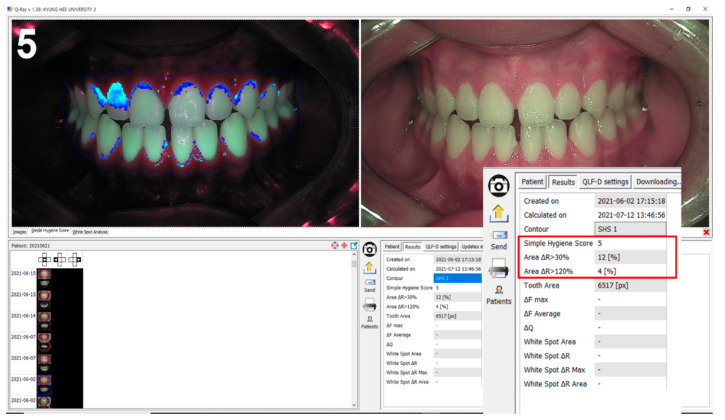
FPI score is automatically calculated with the “Simple Hygiene Score” function of the proprietary analysis program (Q-ray version 1.24, Inspektor Research Systems BV, Amsterdam, The Netherlands).

**Figure 4:**
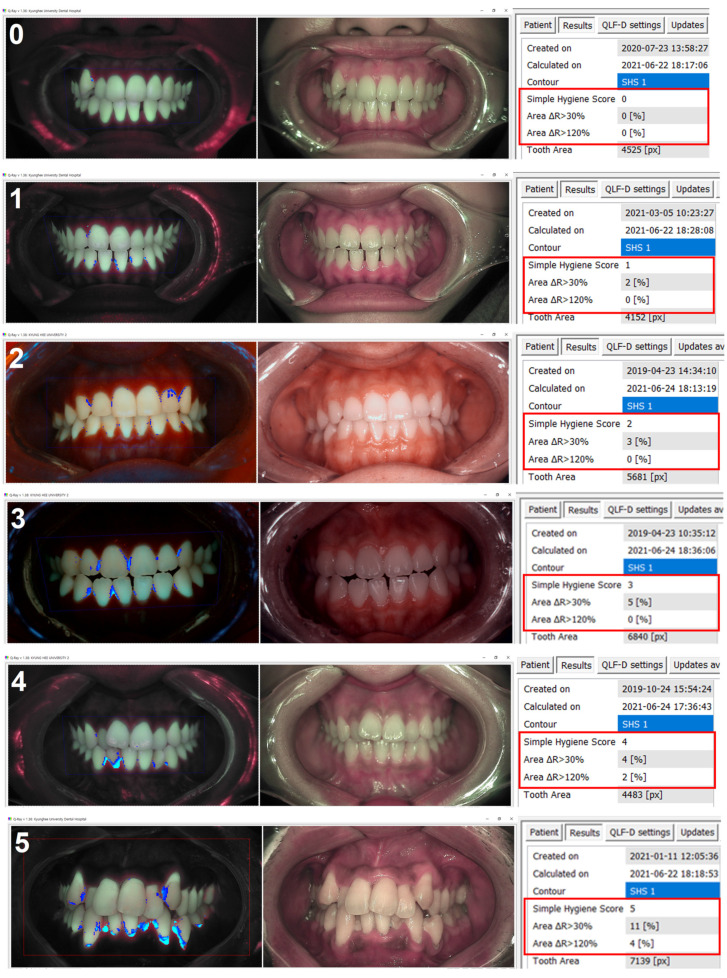
FPI is a score calculated by applying two variables (A_30_ and A_120_), which are regions of red fluorescent plaques corresponding to pixels with ΔR > 30% and ΔR > 120%, to a specific function. FPI scores were calculated in one of six categories (from 0 to 5).

The FPI score for the fluorescent image of the QLF images was automatically calculated with the “Simple Hygiene Score” function of the proprietary analysis program (Q-ray version 1.24, Inspektor Research Systems BV) (Figure 3). FPI was a score calculated by applying two variables (A_30_ and A_120_), which are regions of red fluorescent plaques corresponding to pixels with ΔR > 30% and ΔR > 120%, to a specific function. FPI scores were calculated in one of six categories (from 0 to 5) (Figure 4), according to the classification rules as follows [24]. (1) A_30_ < 0.5 % and A_120_ < 0.4%, FPI score was classified as 0; (2) 0.5% ≤ A_30_ < 2.5% and A_120_ < 0.4% was classified as FPI score 1; (3) 2.5%≤ A_30_ < 4.5% and A_120_ < 0.4% was classified as FPI score 2; (4) 4.5 % ≤ A_30_ or 0.4% ≤ A_120_ < 0.9% was classified as FPI score 3; (5) 0.9% ≤ A_120_ < 2.1% was classified as FPI score 4; (5) 2.1% ≤ A_120_ was classified as FPI score 5. The variable ΔR indicates that porphyrin, a metabolite secreted by bacteria, is present in the oral cavity and assesses the level of bacterial activity.

### 2.4. Radiographic Image Analysis

#### 2.4.1. Acquisition of Panoramic and Periapical Images

The panoramic radiographs were acquired using a dental panoramic X-ray machine (Planmeca Promax^®^, Planmeca, Helsinki, Finland). Periapical radiographs were obtained digitally using a sensor (Kodak RVG 6000, Carestream Dental, Rochester, NY, USA). The radiographs were collected retrospectively after removing identifiable patient information. The radiographic images were assessed using the image analysis tool of the Zetta PACS (Tae Young Soft, Anyang, Korea).

#### 2.4.2. Radiographic Bone Loss (RBL) Scoring

**Figure 5:**
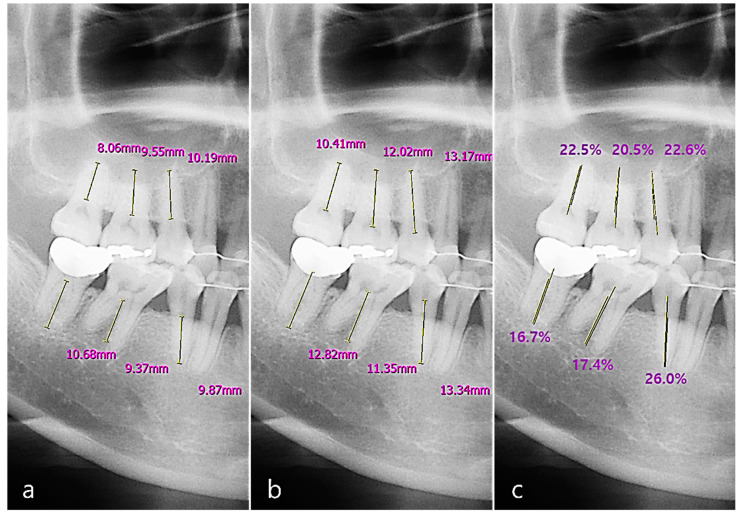
The measurement of RBL (radiographic bone loss) on a panoramic image: (**a**) the distance from the root tip of the tooth to the periodontal bone level (PBL); (**b**) the distance from the root tip of the tooth to the cementoenamel junction level (CEJL); (**c**) the percentage rate of the intersection length of the PBL and the other CEJL.

We used 232 image sets, including both panoramic and periapical images, to detect the periodontal bone level (PBL) and cementoenamel junction level (CEJL) of the teeth. We calculated the two tooth intersection lengths as the distances between the points and the root apex of the tooth. We defined the RBL percentage of the tooth (implant) as the ratio of the intersection length of the periodontal bone level to the CEJ (or the top fixture level for an implant) (Figure 5).

**Figure 6:**
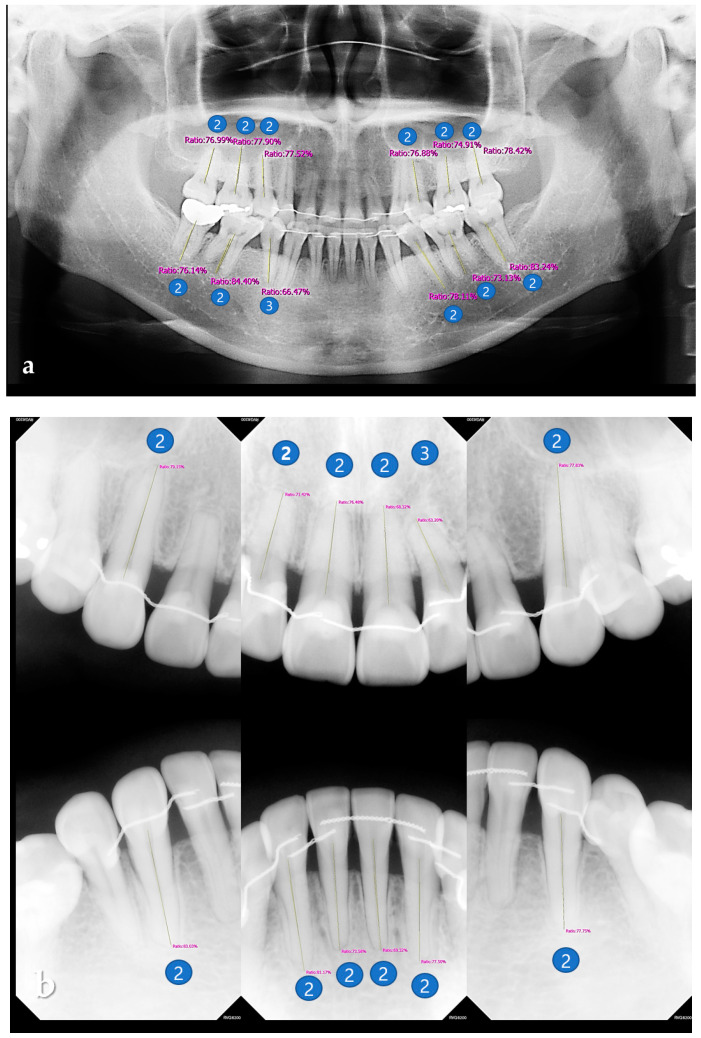
RBL scoring on a panoramic image (**a**) and periapical radiographic image (**b**) based on the percentage rate, to classify the periodontal bone loss of the tooth for periodontitis staging according to the new criteria. The classification criteria were as follows. Score 1: RBL < 15%. Score 2: 15% ≤ RBL ≤ 33%. Score 3: 34% ≤ RBL ≤ 66%. Score 4: RBL > 66%.

Based on the percentage rate, periodontal bone loss of the tooth was classified according to the new criteria proposed at the 2017 World Workshop on the Classification of Periodontal and Peri-implant Diseases and Conditions [16]. The classification criteria for periodontitis staging based on the RBL of the tooth were as follows. (1) RBL < 15% (in the coronal third of the root), the periodontitis was classified as stage one. (2) RBL between 15% and 33% (in the coronal third of the root) was classified as stage two. (3) RBL between 34% and 66% (in the middle third of the root) was classified as stage three. (4) RBL > 66% (extending to the apical third of the root and beyond) was classified as stage four (Figure 6) [16,25]. The average of the RBL scores of the total, maxillary, mandibular, anterior, and posterior teeth were calculated.

### 2.5. Statistical Analysis

The intra-examiner reproducibility of the RBL score was assessed in a second examination of 20 sets of randomly selected teeth two weeks later, in which the Cohen’s kappa value showed significant and excellent agreement (κ values > 0.87). Generalized linear models and regression analyses were used to evaluate the FPI or RBL scores according to oral health habits, sex, and age. The correlations between the FPI and RBL scores were evaluated using Spearman’s correlation coefficient. Statistical analyses were performed using SAS software (version 9.4; SAS Institute Inc., Cary, NC, USA). The results were considered statistically significant at *p* < 0.05.

## 3. Results

Oral health habit was scored out of 12 points. The overall average was 7.52 ± 2.07 (male 6.98 ± 2.20, female 7.88 ± 1.91). The oral health habit score of females was significantly higher than that of males. The FPI distribution showed a value of 1 or less in 78.5% of the patients. The overall average RBL score was 1.50 ± 0.32, and the RBL score did not show a statistically significant difference according to the tooth position (maxilla, mandible, anterior, or posterior) (Table 2).

Oral health habit score, age, and sex showed a statistically significant correlation with FPI. FPI showed a lower value as the oral health habit score increased and the age decreased. Moreover, females showed lower FPI values than did males. RBL showed a statistically significant positive correlation with age but did not show any correlation with oral health habit scores and sex (Table 3 and Table 4).

Spearman’s correlation analysis to examine the mutual effects showed that there was no correlation between FPI and RBL scores (Table 5).

## 4. Discussion

Dental plaque is a complex oral microbial ecosystem that maintains microbial homeostasis through dynamic ecological changes resulting from local environmental conditions [6]. The imbalanced state of the microbial ecosystem results in the formation of pathogenic dental plaques with cariogenic or periodontopathic properties according to the predominant bacteria, their metabolites, and host responses [6,7]. In order to prevent and manage oral diseases, it is necessary to focus on detecting pathogenic dental plaques and predicting the degree of pathogenicity. The optical phenomenon of red fluorescence (RF) used by QLF technology can be explained by the strong fluorescence of endogenous metal-free fluorescent porphyrins such as protoporphyrin IX. It exhibits strong fluorescence in the red spectral region when excited by visible violet light in the 400–420 nm range [26,27]. These porphyrins are produced by certain oral microbes in dental plaque. Previous studies utilizing QLF technology have shown that red fluorescence can be attributed to mature dental plaques. These properties make it easier to detect dental plaque without performing additional staining procedures [14,28]. Compared with non-RF plaques, more periodontopathic bacteria were detected in RF plaques, and it was confirmed that RF plaques were more associated with gingival inflammation [29]. Another previous study found that anaerobic bacteria such as *Fusobacterium, Treponema, Tannerella,* and *Prevotella* increased rapidly in mature dental plaques over 4 days, and that they were associated with gingivitis and periodontitis [30]. As such, it seemed reasonable to infer that the stronger the red fluorescence emitted by the dental plaque is indicative of increased dental plaque maturity and pathogenicity. Therefore, dental plaques with higher red fluorescence intensity can be evaluated as having higher periodontal pathogenicity. It is speculated that the FPI could be used by clinicians to assess the degree of risk for periodontal disease. In this study, no correlation was found between FPI using QLF images and the periodontitis stage using radiographic images. This may be related to the study’s limitations. First, when evaluating pathogenic plaque in the oral cavity of a patient, it is necessary to consider the error caused by assessing only the anterior labial surfaces and using anterior measurements to represent the values of the entire dentition. Therefore, a further study to compare and evaluate the dental plaque index and the degree of radiographic bone loss in each tooth of the dentition should be performed. Second, the clinical indicators of gingivitis were not investigated. Further studies should be performed to evaluate the usefulness of FPI in screening periodontal disease. Radiographic images continue to be regarded as one of the best methods to evaluate alveolar changes associated with periodontitis [16,31,32,33]. However, the radiological findings of periodontal disease in our study were evaluated as the ratio of the root length to the distance between the CEJL and the alveolar ridge without any aids. This subjective assessment might be considered a weak point of diagnosis based on radiological findings. According to Lange [20], it has only limited value in detecting initial RBL through X-ray imaging, and that initial loss of proximal bone tended to either be not detected or be underestimated. [21,34,35,36]. However, according to Ziebioz [36], the measurement of RBL in panoramic images is reproducible and valid, providing reliable results for evaluating the degree of periodontal bone loss in patients with advanced bone loss. In addition, it should be taken into account that X-ray only provides information on bone structure and bone loss and does not reflect current clinical issues [21,22]. It is difficult to make an accurate diagnosis with only X-rays in the case of initial periodontal disease [37]. Therefore, it is necessary to add the results of QLF analysis that can detect pathogenic dental plaque and predict the degree of pathogenicity or questionnaire information that objectively shows oral health habits for a comprehensive diagnosis. Panoramic radiography and periapical radiography have been mainly used to evaluate RBL. In this study, we evaluated RBL using both modalities. The RBL of posterior teeth was evaluated using panoramic images, and the RBL of anterior teeth, which was somewhat distorted in the panorama image, was measured using periapical radiography. Previous study demonstrated that there was a high level of agreement between periapical and panoramic radiographic measurements of the distance between the CEJL and the PBL, as well as the proportional values related to root length [38]. It is time-consuming and labor-intensive to manually measure the RBL of all teeth on panoramic radiographs. Recently, computer-aided diagnosis (CAD) based on deep learning has been widely used to solve complex problems in the field of radiographic interpretation [39]. CAD has been used in oral and maxillofacial radiology to identify caries and periodontal disease, as well as osteoporosis, maxillary sinusitis, and other diseases [40]. A method for automatically diagnosing periodontal bone loss on dental panoramic radiographs for periodontitis staging was developed according to the new criteria proposed at the 2017 World Workshop [16]. This high detection performance of the periodontal bone level (or CEJ level) was achieved by using deep learning to simplify the complexity of bone destruction patterns due to periodontitis [40]. This new strategy can provide a valuable second opinion for dental professionals who need to diagnose periodontitis by automatically detecting and staging pathological changes called periodontal bone loss. This may also substantially improve the diagnosis and treatment of periodontitis [41].

Considering the factors affecting oral health that have been studied so far, not only dietary habits and smoking, but also oral health habits such as dental visits, toothbrushing, and the use of oral hygiene products have been reported as factors affecting oral health [42,43,44,45]. Lifestyle habits account for about 60% of the causes of chronic diseases, highlighting the importance of self-management and professional education programs for correct lifestyle changes [46]. Therefore, for the prevention of periodontal disease, a tool that can measure oral health habits including various risk variables is needed. In this study, a questionnaire was used as a tool to evaluate oral health habits related to periodontal disease, and the results were scored to analyze the effects of oral health habits. As a result, it was shown that the oral health habit score was closely related to the degree of pathogenicity of dental plaque analyzed through QLF images. For individuals with periodontal disease, a high level of oral hygiene is one of the key factors for achieving and maintaining periodontal health [47,48,49]. Therefore, in order to improve individual periodontal disease, professional periodontal treatment is important, but also it is necessary to identify risk factors that may occur in oral health habits, and to change oral health habits in the direction of lowering such risk factors. Educating how to manage these risks in daily life is also necessary. According to Jönsson, based on an integrated cognitive/behavioral and oral health approach, a personalized oral health education program is more effective than standard treatments of reducing plaque and gingivitis by achieving appropriate long-term oral hygiene behaviors [50]. In conclusion, understanding and educating individuals on lifestyle-related risks such as oral health habits, as well as bacterial factors, are necessary for the prevention and treatment of periodontal disease.

## 5. Conclusions

The results of this study suggest that the clinical use of QLF allows plaque detection by non-invasive procedures and can aid in a more objective estimation for oral hygiene status. However, it should be recognized that the presence of bacterial plaque is only one of the factors that influence the development of periodontal disease, and further studies are needed on the bacterial plaque effect on periodontal tissues including alveolar crestal bone.

## Figures and Tables

**Table 1:** Scoring to evaluate oral health habits in patients.

Oral Health Habits	Score
Tooth brushing/day	
None	0
1~2	1
3 or more	2
Tooth brushing before bed/week	
0	0
1~3	1
4~6	2
7	3
Use of oral hygiene products	
No	0
Yes	1
Use of interdental brush or dental floss	
No	0
Sometimes	1
Always use with toothbrush	2
Scaling experience	
None	0
>2 years	1
<2 years	2
<1 year	3
<6 months	4

**Table 2:** Average and classification of oral habit score, FPI and RBL.

**The average of oral health habit score**	Total	7.52 ± 2.07
	Male	6.98 ± 2.20
	Female	7.88 ± 1.91
**The patient number of FPI**	0	125 (53.9%)
	1	57 (24.6%)
	2	6 (2.6%)
	3	16 (6.9%)
	4	15 (6.5%)
	5	13 (5.6%)
**The average of RBL score**	Total	1.50 ± 0.32
	Maxilla	1.50 ± 0.33
	Mandible	1.50 ± 0.36
	Anterior	1.44 ± 0.36
	Posterior	1.56 ± 0.33

FPI: fluorescent plaque index; RBL: radiographic bone loss.

**Table 3:** The multiple generalized linear model estimate between the FPI adjusted by oral health habit score, age and sex.

Variable		Multiple Generalized Linear Model			
		B Estimate	CI		*p*-Value
Oral health habit score	FPI	−0.241	−0.332	−0.150	<0.0001
	A_30_	−0.352	−0.537	−0.167	0.000
	A_120_	−0.122	−0.208	−0.035	0.006
Age	FPI	0.018	0.004	0.032	0.010
	A_30_	0.027	−0.001	0.055	0.051
	A_120_	0.001	−0.012	0.014	0.851
Sex (female)	FPI	−0.458	−0.846	−0.070	0.021
	A_30_	−0.674	−1.463	0.114	0.093
	A_120_	−0.187	−0.555	0.181	0.318

FPI: fluorescent plaque index; A_30_ and A_120_, percentual areas with to tooth area corresponding to all pixels where ΔR over 30 % and 120 %, respectively.

**Table 4:** The multiple generalized linear model estimate between the RBL adjusted by oral health habit score, age and sex.

Variable			Multiple Generalized Linear Model			
			B Estimate	CI		*p*-Value
Oral health habit score	RBL	Total	0.002	−0.017	0.021	0.8707
		Maxilla	−0.002	−0.022	0.019	0.8640
		Mandible	0.005	−0.017	0.027	0.6385
		Anterior	0.005	−0.017	0.027	0.6590
		Posterior	−0.002	−0.021	0.017	0.8245
Age	RBL	Total	0.009	0.006	0.012	<0.0001
		Maxilla	0.008	0.005	0.011	<0.0001
		Mandible	0.010	0.007	0.014	<0.0001
		Anterior	0.007	0.003	0.010	0.000
		Posterior	0.011	0.008	0.014	<0.0001
Sex (female)	RBL	Total	−0.060	−0.141	0.021	0.1477
		Maxilla	−0.058	−0.146	0.029	0.1893
		Mandible	−0.077	−0.170	0.015	0.1018
		Anterior	−0.039	−0.135	0.057	0.4212
		Posterior	−0.084	−0.167	−0.002	0.044

RBL: radiographic bone loss.

**Table 5:** Spearman’s correlation analysis of FPI and RBL scores.

		Coefficient of Correlation	*p*-Value
RBL_Total	FPI	−0.0008	0.9909
	A_30_	−0.0347	0.5996
	A_120_	0.0585	0.3752

FPI: fluorescent plaque index; RBL: radiographic bone loss; A_30_ and A_120_, percentual areas with to tooth area corresponding to all pixels where ΔR over 30 % and 120 %, respectively.

## Data Availability

Data sharing not applicable.
